# Adaptations of *Vibrio parahaemolyticus* to Stress During Environmental Survival, Host Colonization, and Infection

**DOI:** 10.3389/fmicb.2021.737299

**Published:** 2021-10-07

**Authors:** Gururaja Perumal Pazhani, Goutam Chowdhury, Thandavarayan Ramamurthy

**Affiliations:** ^1^School of Pharmaceutical Sciences, Chettinad Academy of Research and Education, Kelambakkam, India; ^2^ICMR-National Institute of Cholera and Enteric Diseases, Kolkata, India

**Keywords:** biofilm, chemotaxis, hemolysin, pathogenicity island, secretion systems, toxin-antitoxin system, *Vibrio parahaemoluticus*, virulence

## Abstract

*Vibrio parahaemolyticus* (Vp) is an aquatic Gram-negative bacterium that may infect humans and cause gastroenteritis and wound infections. The first pandemic of Vp associated infection was caused by the serovar O3:K6 and epidemics caused by the other serovars are increasingly reported. The two major virulence factors, thermostable direct hemolysin (TDH) and/or TDH-related hemolysin (TRH), are associated with hemolysis and cytotoxicity. Vp strains lacking *tdh* and/or *trh* are avirulent and able to colonize in the human gut and cause infection using other unknown factors. This pathogen is well adapted to survive in the environment and human host using several genetic mechanisms. The presence of prophages in Vp contributes to the emergence of pathogenic strains from the marine environment. Vp has two putative type-III and type-VI secretion systems (T3SS and T6SS, respectively) located on both the chromosomes. T3SS play a crucial role during the infection process by causing cytotoxicity and enterotoxicity. T6SS contribute to adhesion, virulence associated with interbacterial competition in the gut milieu. Due to differential expression, type III secretion system 2 (encoded on chromosome-2, T3SS2) and other genes are activated and transcribed by interaction with bile salts within the host. Chromosome-1 encoded T6SS1 has been predominantly identified in clinical isolates. Acquisition of genomic islands by horizontal gene transfer provides enhanced tolerance of Vp toward several antibiotics and heavy metals. Vp consists of evolutionarily conserved targets of GTPases and kinases. Expression of these genes is responsible for the survival of Vp in the host and biochemical changes during its survival. Advanced genomic analysis has revealed that various genes are encoded in Vp pathogenicity island that control and expression of virulence in the host. In the environment, the biofilm gene expression has been positively correlated to tolerance toward aerobic, anaerobic, and micro-aerobic conditions. The genetic similarity analysis of toxin/antitoxin systems of *Escherichia coli* with VP genome has shown a function that could induce a viable non-culturable state by preventing cell division. A better interpretation of the Vp virulence and other mechanisms that support its environmental fitness are important for diagnosis, treatment, prevention and spread of infections. This review identifies some of the common regulatory pathways of Vp in response to different stresses that influence its survival, gut colonization and virulence.

## Introduction

*Vibrio parahaemolyticus* (Vp) is a facultative anaerobic Gram-negative bacterium, belonging to the family *Vibrionaceae*. Serotyping scheme of Vp is based on the antigenic properties of the somatic (O) and capsule (K) antigens, which includes a combination of more than 10 different O and 70 K antigens (Iguchi et al., 1995). This organism is ubiquitous in marine, estuarine ecosystems and a leading cause of seafood-borne diarrheal disease in humans (Li et al., 2019). Vp infected patients typically present with gastroenteritis symptoms such as abdominal cramping, nausea, vomiting, and fever. Patients with comorbidities such as diabetes, liver disease and alcoholism are more likely to develop septicemia. Since its discovery in 1950, this bacterium has been isolated in widespread outbreaks and in sporadic cases of gastroenteritis worldwide (Fujino et al., 1953; Nair et al., 2007). The first pandemic of Vp-associated diarrheal infection emerged in 1996 with a new serotype O3:K6 from Calcutta (now Kolkata), India, which has spread to Asia, Europe, and Americas in the following years (Nair et al., 2007). The number of serovariants all over the world has increased to 49 by 2016 (Han et al., 2016). During 1990–2019, Vp was responsible for more than 40 global outbreaks (Igere and Ekundayo, 2020).

The pathogenicity of Vp depends on a variety of virulence factors. Details of known virulence factors of this pathogen are presented in Table 1. Typically, expression of virulence in Vp is strongly associated with thermostable direct hemolysin (TDH) and/or the TDH-related hemolysin (TRH; Fattel et al., 2019). Stains that lacked *tdh*, *trh*, and T3SS2 caused several diarrheal outbreaks and expressed virulence in animal models (Bhoopong et al., 2007; Chao et al., 2010; Ottaviani et al., 2012; Banerjee et al., 2014; Martinez-Urtaza et al., 2016). However, the exact mechanism is yet to be established.

**TABLE 1:** Details of known virulence factors of *V. parahaemolyticus*.

Effector	Gene/ORF	Activity	Function	References
**Toxins**				
Thermostable direct haemolysin	*tdh*	Pore forming toxin	Haemolytic, cytotoxicity, cardiotoxicity, and enterotoxicity	Raghunath, 2015; Cai and Zhang, 2018
TDH related haemolysin	*trh*	Pore forming toxin	Haemolytic, cytotoxicity, cardiotoxicity, and enterotoxicity	Raghunath, 2015; Nilsson and Turner, 2016
Thermolabile haemolysin	*tlh*	Lecithin-dependent phospholipase activity, and lyses human erythrocytes	Haemolytic and cytotoxicity	Bej et al., 1999; Wang et al., 2012
Multivalent adhesion molecule	MAM7	Binds to fibronectin and phospholipid phosphatidic acid	Facilitates bacterial attachment to host cells by interacting with host cell surface protein fibronectin and plasma membrane phospholipid phosphatidic acid	Krachler et al., 2011
**T3SS1 effectors**				
Vop Q	VP1680	Forms pores and binds V-ATPase	Rapid induction of autophagy, cell lysis, MAPK activation, IL-8 secretion	Burdette et al., 2009; Sreelatha et al., 2013
Vop S	VP1686	Inhibition of Rho family GTPases by AMPylation	Disrupts actin cytoskeleton, cells rounding, phagocytes invasion and actin assembly inhibition	Yarbrough et al., 2009; Luong et al., 2010
VPA0450	VPA0450	Inositol polyphosphate 5-phosphatase	Disrupts plasma membrane integrity, blebbing, destabilization and facilitates cell lysis and participates in cytotoxicity	Broberg et al., 2010, 2011
Vop R	VP1683	Binds PIP2 in membrane	Promoting refolding of T3SS effectors proteins after their delivery into host cell	Wang et al., 2015
**T3SS2 effectors**				
VopC	VPA1321	Activation of Rac and CDC42 by deamidation	Promotes actin nucleation, cytoskeleton, induction of stress fibers, contribute to bacterial uptake into the host cells and invasion of non-phagocytic cells	Zhang et al., 2012; Okada et al., 2014
VopT	VPA1327	ADP-ribosylation of Ras	Induces cytotoxicity and inhibits growth of yeast	Kodama et al., 2007
VopZ	VPA1336	Inhibits TAK1 activation and downstream MAPK and NF-κB	Prevents NF-kB and MAPK signaling, promotes colonization and enterotoxicity	Zhou et al., 2013
VopA/VopP	VPA1346	Inhibition of MAPK by acetylation of MKK	Inhibits MAPK signaling, cell migration, apoptosis, growth of yeast and suppresses the host innate immune response	Trosky et al., 2007
VopV	VPA1357	Actin binding and bundling	Invasion of non-phagocytic cells, causes cytotoxicity and enterotoxicity by fluid accumulation and inhibits growth of yeast	Hiyoshi et al., 2011
VopL	VPA1370	Actin nucleation	Induces actin stress fiber, remodels host cell adherents and tight junction, promotes intestinal colonization, and inhibits growth of yeast cell	Namgoong et al., 2011; Yu et al., 2011
VopO	VPA1329	Polymerize actin	Induces actin stress fiber, remodels tight junction	Hiyoshi et al., 2015
Vpa1380	VPA1380	Cysteine protease	Inhibits growth of yeast cell	Calder et al., 2014
VopW	VPA 1345	Translocate T3SS2 effectors	Promotes colonization and fluid accumulation in rabbit intestine through translocation of T3SS2 effectors	Zhou et al., 2012
VgpB & VgpA	VPA1359 & VPA 1360	Gate way of T3SS secretion	Switches secretion of T3SS2 translocons and T3SS2 effectors on K+ concentration, inhibits growth of yeast	Tandhavanant et al., 2018

Whole genome sequencing of Vp revealed the presence of two sets of type III secretion systems (T3SS; T3SS1, and T3SS2; Matsuda et al., 2020). Of these, T3SS1 gene cluster is ubiquitous in both pathogenic and nonpathogenic strains. Whereas, the T3SS2 gene cluster located in an 80-kb Vp pathogenicity island on the chromosome-2 and has been linked with virulence strains that cause acute gastroenteritis (Okada et al., 2009).

To survive in the environments and in the human host, Vp has several adaptive mechanisms for temperatures, salinity, oxidative stress, and bile salts. Conversion of viable but non-culturable (VBNC) from biofilm formation, quorum sensing regulators, and toxin–antitoxin systems are activated in response to various environmental conditions (Letchumanan et al., 2017; Banerjee et al., 2018). In addition, the intake of prophages and horizontal gene transfer into bacterial chromosomes enhance fitness of the pathogen to encounter several adverse changes in the host (Yang et al., 2020). This review highlights the gaps in our knowledge on how the inherent and acquired factors enhance adaptive mechanisms in Vp that mark the pathogen to survive and enhance the pathogenesis under several stressful conditions.

### Adaptations to Climate Change and Temperature

Adaptation to changing environmental conditions is an important approach for the survival of bacterial pathogens. The combination of climatic and aquatic environmental changes has shaped an ideal condition for the emergence, spread, and resurgence of several infectious diseases, affecting millions of people annually (Deb et al., 2012; Hales, 2019). Many anthropogenic factors, like industrial development, human behavior, and intensive animal farming, have contributed to upsurge in the incidence of infectious diseases (Gage et al., 2008). Of the several factors, the ecosystem variability is most important, as it enhances genomic alternation of some of the pathogenic microorganisms through horizontal gene transfer or mutation, giving rise to new transmission links (Siliakus et al., 2017). Bacteria frequently encounter numerous environmental abiotic stresses (heat, cold, osmotic, salt, oxidation, pH, and radiation) and biotic stresses (antimicrobial compounds and microbial toxins) in their natural life cycle (Beales, 2004). These environmental stresses cause damage to cellular components and inhibit the function of macromolecules that causes bacteria to adapt to varying environmental changes for its survival and proliferation (Brooks et al., 2011; Collin and Hernroth, 2020). Several other defense mechanisms like different growth phases, catalases, and genetic changes can also help them adapt in such stressful environments (Liu et al., 2016; Lovell, 2017). Most of the pathogens equivocate between the mammalian host and natural habitats to utilize the environmental signals to coordinate virulence related gene expression (Thomas and Wigneshweraraj, 2014).

Gastrointestinal pathogens, namely, *Vibrio* spp., *Shigella* spp., and *Yersinia* spp., and pathogenic *Escherichia coli* explore different signals within the human host by expressing several virulence genes. Among these, temperature is one of important activators (Freestone, 2013; Fang et al., 2016). Most of the environmental strains may have conserved virulence-associated mechanisms, but the clinical strains showed a notable difference in response to the human temperature (37°C) such as biofilm formation, motility, and protease production (Mahoney et al., 2010; Hu and Zhang, 2020; Qian et al., 2020). The ecology of Vp is largely influenced by temperature and salinity (Urmersbach et al., 2015). Vp can grow in a wide range of temperature from 16 to 42°C, but the optimum temperature for growth is 37°C (Beuchat, 1982). Rise in the temperature could lead to increase in the abundance of Vp in the environment and therefore could lead to an escalation in the incidence of infections, especially among shellfish consumers during summer months (Yeung and Boor, 2004; Duan and Su, 2005; Chiang and Chou, 2009).

Low temperatures alter gene expression in Vp, for, e.g., the cold-shock proteins (CspA and CspD) are known to protect the Vp at low temperatures (Yang et al., 2009; Zhu et al., 2017). Several stress proteins are formed under the high temperature condition, e.g., heat-shock protein families such as Hsp60 (GroEL and GroES) and Hsp70 (DnaJ, DnaK, and GrpE; Segal and Ron, 1998). These changing temperatures can affect the pathogenicity or the virulence factor expression in Vp (Wong et al., 2002; Chiang et al., 2008). The heat shock causes higher expression of *tdh* (Wong et al., 2002), whereas the T3SS-1 gets downregulated at 15°C and a putative chaperone, hemolytic activity, and the T6SS are upregulated at higher temperatures (Salomon et al., 2013; Urmersbach et al., 2015; Li et al., 2019). The higher temperature also favors an increase in the expression of urease that helps in survival in the gastric acids (Park et al., 2009). The environmental strains that lack the putative virulence genes (*tdh*/*trh*) have the ability to regulate or control differently conserved “virulence-related traits” in response to human body temperature (Mahoney et al., 2010).

### Viable but Non-culturable State

The bacterial cells enter into the VBNC state under extreme conditions, namely, starvation condition and temperature stress (Ramamurthy et al., 2014). Viable but non-culturable state of Vp is likely to cause a food safety threat to public health, as the organism has been identified by the culture-based methods (Fakruddin et al., 2013; Yoon and Lee, 2020). Vp enters into the VBNC state *in vitro* under starvation conditions after 12 days at 4°C or after 30 days in artificial seawater (Mizunoe et al., 2000; Yoon et al., 2017). Under such conditions, Vp shows reduced metabolic activities, losses of colony-forming ability, decreased in ATP synthesis as well as transcription of RNA (Trevors et al., 2012; Jia et al., 2014).

Accumulation of reactive oxygen species (ROS) has been identified as one of the key factors related to the formation of VBNC cells (Cabiscol et al., 2000). Hydrogen peroxide, hydroxyl-free radical, and superoxide anion are the major ROS compounds that enter across the membrane and cell wall under aerobic conditions and degrade the polyunsaturated proteins and fatty acids. This condition causes a reduction in membrane fluidity, which enhances the formation of VBNC cells (McDougald et al., 2002). Vp can develop specific survival mechanisms using several antioxidant defense systems against ROS, including alkyl hydroperoxide reductase (AhpC), catalase, and peroxidase (KatG; Lai and Wong, 2013). Using suitable stimuli in the medium supplemented with catalase or sodium pyruvate (H_2_O_2_-degrading compounds), the VBNC cells have been resuscitated to culturable cells (Mizunoe et al., 2000).

Interestingly, the VBNC state of Vp has shown the ability of adhesion and virulence expression under *in vitro* conditions (Chiang et al., 2005; Tang et al., 2018). In addition, the VBNC state of Vp exhibited the strong cytotoxic activity to HEp-2 cell lines and able to colonize *in vivo* (Baffone et al., 2003; Wong et al., 2004). Vp can develop specific survival mechanisms using several antioxidant defense systems against ROS, namely, alkyl hydroperoxide reductase (AhpC), catalase, and KatG (Lai and Wong, 2013).

### Adaptations to Salinity, Oxidative, and Ethanol Stresses

Vp can survive varying concentrations of NaCl (0.5%–10.5%) for their growth and maintain an osmotic balance with their external environment. The optimal concentration of 3% NaCl has been used in the different media for the growth and isolation of this *Vibrio* (Wong and Wang, 2004). To support the cell stability in the presence of high salinity concentrations, Vp has different halophilic proteins, characterized by a large number of acidic amino acids, negatively charged with hydrated carboxyl groups, and less in hydrophobic amino acids (Ongagna-Yhombi and Boyd, 2013; Gregory and Boyd, 2021). Lysine decarboxylase (encoded by *cadA*) is another acid-resistance transcriptional system that has been well-characterized in enteric pathogens (Neely and Olson, 1996; Merrell and Camilli, 1999). Under acidic conditions, *cadA* has induced the adaptation of the amino acid lysine to the basic product cadaverine, which increases the hydroxide ions (Soksawatmaekhin et al., 2004). In Vp, *cadA* is responsible for decarboxylation of lysine and transcriptional expression of the lysine decarboxylase in the presence of external lysine (Tanaka et al., 2008). In Vp, the *cadA* expression was significantly higher in a minimal medium with 3% NaCl than with 1% NaCl (Whitaker et al., 2010). The importance of *cadA* was further demonstrated in Vp mutant strain, which had a shorter survival in high saline condition (Kalburge et al., 2014).

RNA polymerase, which is a sigma S (RpoS) factor, generally regulates various genes and enhances cross-protection against several environmental stresses (Stokes et al., 2003). RpoS is important for cell survival under oxidative and acidic stress conditions and also controls the expression of mechanosensitive channels in different enteric bacteria including Vp (Vasudevan and Venkitanarayanan, 2006; Tan et al., 2010). Expression of *rpoS* in Vp is generally higher to support the survival in the high saline conditions. *rposS* mutant stain exhibited significantly decreased resistance when grown in 3% NaCl, indicating the importance of this gene’s function (Huang and Wong, 2012). *rpoS* also plays a crucial role in the survival of Vp under the stressed conditions of cold and hyperosmolarity (Whitaker et al., 2010). Vp uses catalases, alkyl hydroperoxide reductases, antioxidative enzymes, and antioxidative encoding genes, which protect against different oxidizing agents such as hydrogen peroxide (H_2_O_2_), peracetic acid (C_2_H_4_O_3_), and chlorine dioxide (ClO_2_; Chen et al., 2016; Yu et al., 2016). These oxidizing agents are commonly used in food and health care industries to inactivate vegetative cells and spores of food-borne pathogenic bacteria and for the treatment of fresh produce, seafood, and ready-to-eat food (Leggett et al., 2015; Tso et al., 2019). Several genes encoding key antioxidant enzymes, namely, *katE*, *oxyR*, *rpoS*, and *ahpC*, protect Vp from oxidizing agents (Chen et al., 2016; Yu et al., 2016).

Ethanol is commonly used as a food preservative or to disinfect microorganism from the surface of utensils and equipment to maintain a hygienic environment in food processing industries. However, microorganisms significantly increased their resistance to ethanol after adaptation to a sublethal dose of ethanol (Chiang et al., 2006). Vp resistance up to a concentration of 8% ethanol at 47°C. Under this condition, several changes have been observed in Vp including cell morphology, expression of a different protein, catalase, higher expression of *tdh*, changes in the fatty acid profile, and increased susceptibility to high salt, crystal violet, and organic acid stresses (Chiang and Chou, 2008; Chiang et al., 2008).

### Adaptations to Bile Salts

Cholic and chenodeoxycholic acid are the primary bile acids synthesized in the liver, which are conjugated to glycine or taurine before secretion. The colonic bacteria in the intestine covert the primary bile acids to the secondary bile acids (deoxycholic acid and lithocholic acid). Bile salts are needed in the human gastrointestinal tract to support break down of fats, aid digestion, absorb vitamins, and inactivate bacterial toxins (Begley et al., 2005; Sistrunk et al., 2016). In addition, bile salts also prevent colonization of pathogenic bacteria in the gastrointestinal tract (Urdaneta and Casadesús, 2017). Enteric pathogens sense bile as an environmental cue to control or regulate their virulence factors by decreasing membrane permeability, inducing biofilm formation and efflux pumps, and upregulating redox and DNA damage repair genes (Gunn, 2000; Merritt and Donaldson, 2009).

Vp uses bile salts as an environmental signal to upregulate virulence genes during infection (Rivera-Cancel and Orth, 2017). In the gut, the presence of conjugated bile acids enhances the expression of *tdh* and activation of T3SS2 systems, which consequently increases the enterotoxicity with acute gastroenteritis (Osawa and Yamai, 1996; Letchumanan et al., 2017). In the presence of bile salts, Vp uses inner-membrane proteins, VtrA, VtrB, and VtrC, which activate the T3SS2 (Li et al., 2016). The highly conserved VtrA/VtrC form a 1:1 protein complex through their periplasmic domains to form a membrane-bound receptor and activates VtrB on the surface of the membrane to induce the expression of T3SS2-related genes through the Vp pathogenicity island (PAI) promoters (Letchumanan et al., 2017). The protein complex creates a barrel-like structure that can bind to bile salts and trigger the cell to produce toxins.

### Role of Biofilm and Quorum Sensing Regulators for Adaptations

Expressions of virulence-associated genes in Vp depend on the bacterial cell density. This phenomenal change has been recognized as Quorum Sensing (QS), which is very well-documented in most of the vibrios. Quorum Sensing regulates the gene expression with respect to changes in the bacterial cell density by autoinduction of various genes. At a low-cell density level of vibrios, Sigma 54-dependent factor regulates genes such as *aphA* and *opaR* (Kalburge et al., 2017). The role of *aphA* and *opaR* is important in the gut colonization of Vp. LuxR family transcriptional regulator *aphA* is important for the expression of *opaR* and the regulation of lateral flagella (Lu et al., 2019). Double mutant of *aphA* and *opaR* genes affects gut colonization of Vp *in vivo* (Kalburge et al., 2017). OpaR controls metalloprotease, serine, and protease genes that regulate environmental survival and bacterial virulence (Chang and Lee, 2020). An extracellular serine protease encoding gene *prtA* involved in nutrient uptake as well as hemolytic and cytotoxic activities is required for establishing infection (Chang and Lee, 2018).

The quorum sensing synthase gene *cqsA*, which corresponds to VPA0711 in Vp strain RIMD2210633 genome, has been reported to give the signal through 3-hydroxyundecan-4 one molecule that regulates colony morphology and upregulation of another QS-associated gene *opaR* (Wu et al., 2019). At a low cell density of Vp in the environment and host, nitric oxide (NO) activates the master QS regulatory gene *opaR*. Nitric oxide is associated with the bacterial life cycle by involving an active role in the regulation of biofilm production, metabolism, and singling pathways of cyclic di-GMP (Ueno et al., 2019). This cyclic di-GMP is programmed by a capsular polysaccharide (CpsQ), which is part of the membrane fusion protein (MFP). The protein-encoding MFP locus is required for biofilm production in Vp (Zhang et al., 2021).

The *lux* operon encodes various genes, which are self-regulated and produce luminescent proteins. This was originally discovered in *Vibrio fishceri*. *luxM* and *luxS* are implicated in the adaptation of Vp in various niches (Guo et al., 2018). At high concentrations, due to the presence of *lux* operon, Vp produces a biomolecule called autoinducer (AI, 4-hydroxy-5-methyl-3(2h)-furanone and naphthalene derivatives), which supports QS-mediated regulation of biofilm formation and virulence (Vendeville et al., 2005; Mizan et al., 2017). Swarming is important for Vp to grow on different surfaces. A three-gene operon (*scrABC*) has been identified for swarming effect, which encodes a pyridoxal-phosphate-dependent enzyme, an extracellular solute-binding protein, and a membrane-bound GGDEF- and EAL-motif sensory protein. These genes may control up- and downregulation of lateral flagellar gene expression and capsular polysaccharide (CPS) production in Vp. In addition, a gene encoding a diguanidylate cyclase/phosphodiesterase GGDEF-EAL domain protein inversely regulates the swarming effect and CPS production in bacteria by modulating small signaling nucleotide cyclic di-GMP (Boles and McCarter, 2002; Trimble and McCarter, 2011). An omics-based study identified a hypothetical protein, VP0610, which acts on bacterial phosphotransferase and QS systems and that yield several phenotypical changes in Vp such as biofilm formation, swimming motility, and swarming effect (Jiang et al., 2021).

## Horizontal Gene Transfer and Bacterial Fitness

**FIGURE 1:**
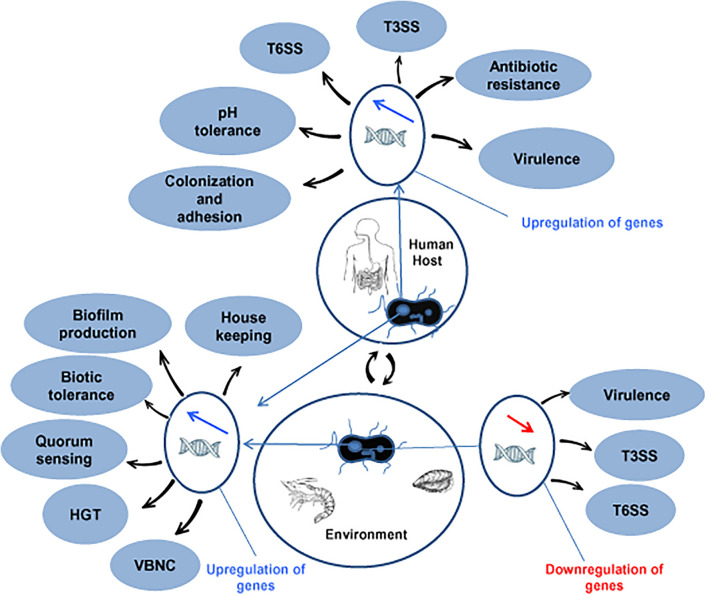
Up- and downregulated in genes in *V. parahaemolyticus* for adaptation and fitness in human and environmental niches. HGT, horizontal gene transfer; VBNC, viable but non-culturable; T3SS, type III secretion system; T6SS, type VI secretion system.

Vp causes diarrhea in humans and several infections in fish/shrimps. Horizontal Gene Transfer (HGT) in Vp make changes in serovars and provide several functional benefits including antimicrobial resistance. HGT make alterations in the genome of Vp by making modifications in the total G+C content, location of superintegrons and prophages and thereby facilitating the emergence of pandemic strains of Vp (Espejo et al., 2017). The presence of acquired genes helps this bacterium to adapt to various conditions in humans and the marine environment (Makino et al., 2003). Some of the genes that acquired thorough HGT support the regulation of ToxR and many of them are involved in the up- or downregulation either in the human host or environment (Figure 1). In the aquatic environment, chitin supports natural transformation of genetic materials in Vp. Overexpression of the master regulator TfoX occurs in the presence of chitin, which allows the natural transformation of Vp (Chimalapati et al., 2018). The serovar O3:K6 has been recognized as the first pandemic strain and from which other serovars, like O4:K68, O1:K25, O1:KUT, and O6:K1, are emerged and spread in several countries. The whole-genome analysis of these serovariants has shown several recombination events like insertion and duplication genetic traits in the lipopolysaccharide and capsular polysaccharide loci via HGT (Espejo et al., 2017). Capsular modification is beneficial for bacterial persistence, adaptation to diverse environmental conditions, and preventing bacteria from physical and chemical stress in various niches (Klein et al., 2018; Rendueles et al., 2018).

Pathogenicity island (PAI) and most of the AMR encoding genes in Vp are acquired mostly by HGT. AMR encoding genes that correspond to resistance to ampicillin, streptomycin, amikacin, kanamycin, tetracycline, chloramphenicol, and colistin have been reported in Vp (Li et al., 2014; Elmahdi et al., 2018; Jiang et al., 2019). Resistance to third-generation cephalosporins have also been reported in Vp isolated from shrimps (Liu et al., 2013; Letchumanan et al., 2015).

**FIGURE 2:**
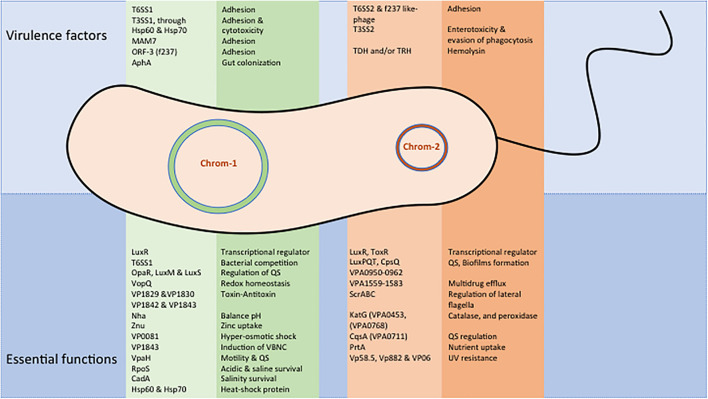
Major virulence and essential functional proteins of *V. parahaemolyticus.* Expressed gene functions are shown separately for chromosome-1 and 2.

Transferable conjugative plasmids are responsible for the transmission of most of these AMR determinants (Liu et al., 2013; Han et al., 2015). In addition, Vp develops mutations in the chromosome to confer resistance to fluoroquinolones (Lei et al., 2020). Some of the genes transferred into the PAI and transposons help in the integration of foreign DNA into the genome of Vp. In the genomic island of Vp, a Na+/H+ antiporter encoding gene *nhaA* has shown to transport ions across the membrane to balance the pH (Klein et al., 2018). Zinc is an important micronutrient required for bacterial metalloenzyme activation, intracellular invasion, survival, and replication within the host. In Vp, horizontally acquired gene *zunA* has been identified, which helps in zinc uptake through ZnuACB transportation and provides better adaptability of Vp in the new environment (Liu et al., 2013). The genes encoded on the chromosome-1 and 2 of Vp express several essential functional proteins (Figure 2). The horizontally acquired gene *vpaH* expresses a histone-like nucleoid structure (H-NS), which regulates the biosynthesis of lateral flagella and supports motility and QS (Park et al., 2005). Genomic analysis of Vp has also shown a set of horizontally acquired gene encoding the T3SS and toxin–antitoxin functions (Ramisetty and Santhosh, 2016).

## Prophages Supports Survival and Adaptations

A comparative genomic analysis of environmental and clinical strains identified several types of prophages and the majority of them were found in environmental strains. Bacteriophages are known to infect bacteria and hijack various cellular machinery of the host. Phages are self-replicating, self-limiting, and resist secondary metabolites produced by the bacteria. Many of these products are beneficial to the host by enhancing the fitness to survive in the different environmental conditions (Wendling et al., 2021). Most of the marine viruses identified to have bacteriophages, which play a crucial role in controlling bacterial mortality, gene expression, and also promote the horizontal gene transfer (Zabala et al., 2009; Simmons et al., 2018). The prophages such as Vp58.5, Vp882, and VP06 in Vp enhance the function of ultraviolet sensitivity, DNA methylase, and quorum sense and resist environmental stress, respectively (Lan et al., 2009; Zabala et al., 2009; Wong et al., 2019). Strain-specific Martha12B12 phage that encode a hypothetical protein-encoding gene VpaChn25_0724 was found to not only influence bacterial growth, motility, biofilm formation, production of secretomes, and also protects bacteria against host phagocytosis (Yang et al., 2020).

Prophages not only determine the evolutionary tread of Vp but also help the bacterium in its normal function and expression of virulence. The presence of the phage VfO3K6 in Vp was found to be associated with acute hepatopancreatic necrosis disease in shrimps (Yu et al., 2020). The epidemic strength of Vp has been correlated with the acquisition of specific open reading the frame (ORF)-8 by the infective phage f237. The ORF-8 protein has been reported for adhesion of Vp in the host intestine and also the surface of marine plankton (Nasu et al., 2000). A clinical non-toxigenic Vp strain isolated from Chile was found to have a prophage-like element that was similar to f237 phage that encoded gene for the Zonula occludens toxin (Pérez-Reytor et al., 2020). This toxin has been recognized as an important factor for *Vibrio cholerae* for intestinal permeation in the mammalian host cell and destabilization of the cytoskeleton of the host cell (Fasano et al., 1995). As a defense mechanism, bacteria have the clustered regularly interspaced short palindromic repeats (CRISPR) along with Cas proteins, which damage the DNA from similar bacteriophages during subsequent infections (Deveau et al., 2010).

## Functions of Toxin–Antitoxin System

Toxin–Antitoxin (TA) is a set of closely linked genes that collectively encode both a “toxin” protein and a corresponding “antitoxin.” Toxin–antitoxin systems are classified into several types based on how the antitoxin neutralizes the toxin. Toxin–Antitoxin system may be located in the plasmid or chromosome of bacteria and contributes to virulence and bacterial fitness in different environmental conditions. Some of the chromosomal TA systems are associated with the cell functions such as responding to stress and causing cell cycle arrest (Van Melderen and Saavedra De Bast, 2009). When the bacterial cells are under stress, the TA system supports induction of VBNC state (Hayes and Low, 2009).

Genomic analysis of Vp strain RIMD2210633 has shown two gene clusters, vp1829/vp1830 and vp1842/vp1843, which had homology with *E. coli* TA system that encoded for DinJ/YafQ, comprising the DinJ antitoxin protein/ribosome-dependent RNase YafQ toxin (Yamaguchi and Inouye, 2011). These gene clusters are responsible for bacterial cell life at natural and different environmental conditions. The gene cluster of vp1842/vp1843 in Vp was reported to locate within a superintegron of chromosome-1, which is involved in cell growth and regulating the morphology of bacteria (Zhang J. et al., 2017). The gene vp1843 was expressed in *E. coli* and identified to inhibit cell growth by halting the cell division by the induction of chromosomal DNA degradation (Zhang J. et al., 2017) and to cause the induction of VBNC state in Vp (Hino et al., 2014; Zhang J. et al., 2017). The gene cluster of vp1842/vp1843 in Vp was found highly conserved in most of *Vibrio* species and that was identified to induce the VBNC state (Hino et al., 2014). Further, Vp1843 has been identified in the part of RelE/ParE toxin superfamily to regulate multiple functions in the bacteria like protein synthesis inhibitory activity and ribonuclease activity. Through TA mechanism, environmental Vp could acquire virulence plasmid genes encoding PirAvp/PirBvp and translate into Cry insecticidal toxin-like proteins with pore-forming activity (Lee et al., 2015). Death on curing (Doc) and prevent host death (Phd) encoding genes were identified in the TA systems in many vibrios. Multiple copies of these genes provide fitness to the PAI in Vp (Klein et al., 2018).

## Function and Regulation of Pathogenicity Islands

The TDH and TRH encoding genes are regulated by ToxR, which is a membrane-localized regulatory protein that plays an essential role in the expression of virulence and modulating bacterial persistence. ToxR is commonly present in most of the *Vibrio* species, but sequence similarities of this gene in Vp and other vibrios remain less than 60% (Matz et al., 2011). Based on the nucleotide constitute, five alleles of TDH and two alleles of TRH have been reported in clinical Vp strains. These toxins are important for the establishment of disease in the host and to cause gastroenteritis and diarrhea. The gene *tlh* encode thermolabile hemolysin, which is a phospholipase A2. However, the contribution TLH to Vp pathogenicity is unknown (Zhang and Austin, 2005). Production of hemolysin has been correlated with bacterial cell density. Decreased hemolysin production was reported with an increased cell density of Vp (Mahoney et al., 2010). In other bacterial pathogens, hemolysin secretion and functions are different and also depended on the bacterial cell density. At higher concentrations, it can lyse erythrocytes, kill epithelial cells, and damage leukocytes by inducing pores. At low concentration, hemolysin is able to alter its own cellular functions and induce host proteases, especially mesotrypsin that cause detachment of cell, cell membrane damage, and cell death (Hongwei et al., 2021).

T3SS and T6SS are encoded on the genomic islands of both clinical and environmental strains of Vp. T3SS is acquired by HGT and present in both the chromosomes of Vp as T3SS1 and T3SS2. The genes encoded in the T3SS1 express several proteins responsible for bacterial survival in the different environmental conditions by regulating biofilm production, motility, and cytotoxicity. T3SS1’s VopQ effector protein control host metabolic processes like a glycolytic, tricarboxylic acid cycle, and amino acid and also alter the host cell redox homeostasis (Nguyen et al., 2020). Genes encoded in the T3SS2 involve in the negative regulation of cellular inflammatory response, which supports bacterial survival in the host through evasion of phagocytosis (Li et al., 2019). The gene *VPA0226* encoded on T3SS2 secretes lipase in the host cell cytoplasm, which is indirectly used to esterify cholesterol that allows the bacteria to escape from the cell by damaging the plasma membrane (Chimalapati et al., 2020). Collectively, T3SS effector proteins support Vp to reside, propagate within a vacuole, and able to adopt a customized intracellular lifestyle within the cytosol of a broad range of host (De Souza Santos and Orth, 2019). The function of T3SS seems niche specific. In oysters, the persistence of Vp is due to the expression of type 1 pili, type IV pili, and flagellar system, which are not associated with T3SS (Aagesen et al., 2013). Similar to T3SS, T6SS are tightly regulated by external factors and deliver several proteins that help the pathogen to compete with other bacteria populations and provide better fitness to survive in the environment (Ben-Yaakov and Salomon, 2019; Fridman et al., 2020). In addition, the effector proteins of T6SS induce the pathogenesis in the host cell by enhancing the mammalian cell adhesion, provoke an autophagy response in the macrophages, and regulate the QS (Yu et al., 2015; Zhang Y. et al., 2017).

## Summary and Conclusion

Vp is a marine pathogen that causes gastroenteritis due to consumption of contaminated raw or undercooked seafood. Wound infections/septicemia produced by this pathogen is mainly due to exposure to coastal waters and a weak immune system. Vp is influenced by several factors in the marine environment and in the human host. Some of the important environmental factors such as inorganic pollutants, heavy metals, salt concentrations, temperature, changes in the pH, and nutritional availability play a crucial role in the life history and ecology of Vp. This pathogen has several genetic mechanisms to adapt into various such challenges that threat its survival. Quorum sensing and biofilm formation are the two important adaptive features commonly used by the vibrios. Exposer of Vp under stressed conditions lead to activation of various gene regulation systems for conversion of normal cells into VBNC state. In the environment, Vp uses several aquatic animals like shrimp, crab, and oysters to access growth factors such as amino acid, lipids, vitamins, and minerals to keep the cells metabolically active until it enters into the host. In addition, HGT of catabolic system encoding genes support the bacteria for its enhanced survival and fitness in different niches. In the host, ToxR regulation stimulates secretion systems and other virulence genes. Though the basic process of Vp associated infection is known, there is a paucity of information on how the pathogen initiates the infection in the host using different gene machinery and host cell response. Hence, there is a need for more detailed molecular/functional studies coupled with genomic analysis to understand pathogenesis of Vp.

## Author Contributions

GP and GC were equally contributed in writing the manuscript. TR corrected and edited the manuscript. All authors contributed to the article and approved the submitted version.

## Conflict of Interest

The authors declare that the research was conducted in the absence of any commercial or financial relationships that could be construed as a potential conflict of interest.

## Publisher’s Note

All claims expressed in this article are solely those of the authors and do not necessarily represent those of their affiliated organizations, or those of the publisher, the editors and the reviewers. Any product that may be evaluated in this article, or claim that may be made by its manufacturer, is not guaranteed or endorsed by the publisher.
